# The Effects of Utilizing Cartilage Conduction Hearing Aids among Patients with Conductive Hearing Loss

**DOI:** 10.3390/audiolres13030036

**Published:** 2023-06-01

**Authors:** Takuya Kakuki, Ryo Miyata, Yurie Yoshida, Aya Kaizaki, Ayami Kimura, Kaede Kurashima, Rui Kuwata, Kenichi Takano

**Affiliations:** 1 Department of Otolaryngology-Head and Neck Surgery, Sapporo Medical University School of Medicine, Sapporo 060-8556, Hokkaido, Japan; y-yurie@sapmed.ac.jp (Y.Y.); kent@sapmed.ac.jp (K.T.); 2 Ebetsu City Hospital, Ebetsu 067-8585, Hokkaido, Japan; ryo.miya@sapmed.ac.jp

**Keywords:** cartilage conduction, hearing aid, conductive hearing loss, speech recognition

## Abstract

The cartilage-conduction hearing aid (CC-HA) is a new hearing device that is suitable for use in patients with conductive hearing loss. It has been 5 years since the introduction of the CC-HA. Although the number of users has increased, the CC-HA is not yet widely known. This study examines the effects of CC-HA on patients with conductive hearing loss and investigates factors that affect the willingness to use the device by comparing purchasers and non-purchasers of CC-HA in patients with unilateral conductive hearing loss. Eight patients had bilateral conductive hearing loss, and 35 had unilateral conductive hearing loss. Each patient underwent sound field tests and speech audiometry, and the effects of the CC-HA were compared with those of conventional bone conduction hearing aids (BC-HA). In patients with bilateral conductive hearing loss, the CC-HA was non-inferior to BC-HA. The CC-HA improved the hearing thresholds and speech recognition in patients with unilateral conductive hearing loss. Moreover, in patients with unilateral conductive hearing loss, experiencing the effect of wearing the CC-HA under conditions such as putting noise in the better ear could affect patients’ willingness to use the CC-HA.

## 1. Introduction

It has long been postulated that auditory sound conduction is facilitated solely through two pathways: air conduction (AC) via the ear canal and bone conduction (BC) through the skull. However, in 2004, Hosoi [1] discovered that the application of vibrations containing sound information to the cartilage produces a sound that is as clear as air or bone conduction. This phenomenon was termed “cartilage conduction” (CC). CC was subsequently established as the third auditory pathway following air and bone conduction.

Three sound conduction pathways are now believed to exist from the CC transducer to the inner ear: the vibration of the ear cartilage produces air-conducted sound in the external auditory canal, which reaches the tympanic membrane; it is referred to as “cartilage AC”. Vibrations of the otocardium generate vibrations in the temporal bone, which are transmitted to the inner ear, which is known as “cartilage BC”. The CC transducer vibrates the air surrounding it, and the resulting air-conducted sound enters the external auditory canal through its entrance and reaches the tympanic membrane. This is called “direct AC”. These three pathways have been previously described [2,3].

The concept of CC has been utilized in the development of products such as hearing aids, smartphones, and earphones [4]. Specifically, the development of cartilage-conduction hearing aids (CC-HA) [5,6,7] that were introduced in 2017 has progressed rapidly.

Conventional sound conduction methods for hearing aids typically rely on air conduction. A condition that is difficult to manage with an air-conduction hearing aid (AC-HA) is aural atresia (e.g., microtia). In such cases, the use of AC-HA can be difficult or ineffective due to the closed outer ear. In these patients, bone-conduction hearing aids (BC-HA), implantable bone-conduction hearing aids (BAHA, BONEBRIDGE), and middle ear implants (VSB) have been employed. BC-HA is bone conductive; therefore, sound conduction is not significantly affected even if the ear canal is closed. However, a headband must be used to attach the transducer to the bone to achieve optimal hearing. However, prolonged use is challenging because of local pain, indentation, redness, and erosion caused by headband fixation, which is also aesthetically unappealing. BAHA, BONEBRIDGE, and VSB can address the limitations of bone-anchored hearing aids because they are surgically implanted. However, the requirement for surgery is a significant drawback [8,9,10]. The CC-HA is suitable for use even with microtia, as conduction occurs through vibrations in the ear cartilage, and it is painless because the transducer does not need to be firmly clamped to the body, as in the case of BC-HA. The CC-HA does not require a headband for fixation, and the transducer can be easily secured. Additionally, the CC-HA does not require surgery. In summary, the lightweight and compact CC-HA transducer is more comfortable and aesthetically pleasing than the BC-HA [11,12,13,14,15]. In CC-HAs, the degree of contribution to the above three conduction pathways changes according to the frequency of sound and the condition of the outer and middle ears, meaning it is difficult to simplify their effects. For example, the cartilage-AC route is the main route in an ear with a normal ear canal, whereas the cartilage-BC route is the main route in an ear with aural atresia. CC-HAs are, therefore, different from conventional AC and BC-HAs, and there are cases where they have merit over conventional hearing aids if these characteristics can be utilized.

Nishimura et al. [12] reported the benefits of CC-HAs in patients with severe conductive hearing loss and concluded that the functional gains for CC-HAs were nearly equivalent to that for previously used hearing aids, with 39 out of 41 patients continuing to use CC-HAs instead of their original hearing aids. Nishiyama et al. [13] assessed the efficacy of CC-HAs in 37 adult patients with hearing loss who had various anatomical conditions in their ear canal, and they concluded that adult patients with ear canal stenosis or closure were the best candidates for CC-HAs, regardless of their hearing thresholds. Nishiyama et al. [14] also reported the efficacy of CC-HAs in 42 pediatric patients with hearing loss, and they concluded that CC-HAs were efficacious in producing hearing improvements in children, especially in patients with atresia or canal stenosis who could not use AC-HAs.

It has been 5 years since the introduction of the CC-HA, and although the efficacy of the CC-HA, such as those mentioned above, has been reported, it is not yet widely used worldwide. In this study, we examined the effect of wearing the CC-HA on patients with conductive hearing loss at our hospital who had a hearing aid trial with the CC-HA.

## 2. Materials and Methods

Participants included eight patients with bilateral conductive hearing loss and 35 patients with unilateral conductive hearing loss who underwent a hearing aid trial with the CC-HA at our hospital (Sapporo Medical University) over a period of 5 years from December 2017 to December 2022. Among patients with bilateral conductive hearing loss, we included patients who were using BC-HAs as their conventional hearing aid and excluded patients who were not using BC-HAs. Patients with bilateral conductive hearing loss were 6–27 years of age (median 13 years) and consisted of two males and six females, whereas those with unilateral conductive hearing loss were 4–64 years old (median 15 years), consisting of 20 males and 15 females. Among the patients with bilateral conductive hearing loss, six had microtia, and two had external auditory canal stenosis or atresia with a normal concha. Of the patients with unilateral conductive hearing loss, 30 had microtia, three had external auditory canal stenosis or atresia with a normal concha, one had a middle ear malformation, and one had undergone surgery for external auditory canal cancer.

For the hearing aid trial, from December 2017 to October 2020, HB-J1CC (Rion Co., Ltd., Kokubunji, Japan) was used, and from October 2020, HB-A2CC, a new model of the same device, was used. The transducers selected were ear-chip-embedded or simple types according to the ear condition. Since HB-J1CC does not have a child lock function, it was said to be suitable for ages 3 and up. However, the HB-A2CC was equipped with a child lock function to prevent the accidental swallowing of the battery, allowing for its use from a younger age.

Simple pure-tone audiometry, sound field tests, and speech audiometry were performed in patients with bilateral conductive hearing loss. Regarding speech audiometry, two patients did not consent to participate, and speech audiometry was not performed. For the sound field test and speech audiometry, the test results obtained when using conventional BC-HA were compared with those obtained using the CC-HA. For patients with unilateral conductive hearing loss, we performed simple pure-tone audiometry, sound field tests, and speech audiometry. Similar to the method described by Akasaka et al. [16] to examine the effect of binaural hearing with the CC-HA in unilateral aural atresia, from December 2017 to September 2019, we compared speech recognition scores (SRS) with and without the CC-HA at sound pressure 10 dB lower than the sound pressure at which the highest SRSs were obtained without a hearing aid. After October 2019, we conducted a sound field test with 70 dB noise (narrow band noise) from headphones worn on the better ear with and without the CC-HA and compared the results to further clarify the effect of wearing the CC-HA. The noise was set at 70 dB to avoid excessive discomfort to the better ear, but in some patients with a high threshold on the affected ear, sound field hearing without the CC-HA may have been incorrect. Similarly, we compared the SRSs conducted at a presentation sound pressure of 60 dB with 70 dB noise (speech noise) in the better ear with and without the CC-HA. One patient did not cooperate with the sound field test, and four patients did not cooperate with the speech audiometry. The results of the pure-tone audiometry and sound field tests were averaged at thresholds of 500, 1000, and 2000 Hz. Speech audiometry was performed using the 57-S list and 67-S list authorized by the Japan Audiological Society. The 57-S or 67-S word lists included 50- or 20-monosyllable words, respectively. For a detailed evaluation, it was better to use the 57-S list, which had a large number of monosyllables but took a long time to examine. Therefore, the 67-S list was used when conducting speech audiometry to obtain the highest standard SRS without hearing aids. On the other hand, the 57-S list was used in the speech audiometry to compare the difference with and without the CC-HA. Pure tone audiometry, sound field testing, and speech audiometry were all performed in a soundproof room. In the sound field test and speech intelligibility test, speakers were located at a distance of 1 m from the patient and at an angle of 45 degrees to the left and right.

Welch’s *t*-test and a chi-squared test were used for statistical analyses. Analyses were performed using Microsoft 365 Excel (Microsoft Co., Redmond, WA, USA), and statistical significance was set at *p* < 0.05.

We revealed the purpose and content of the survey to the participants and ensured the protection of privacy. This study was approved by the Ethics Committee of Sapporo Medical University in an official letter on 27 February 2023 (Protocol number: 342-232).

## 3. Results

The clinical characteristics of the study participants are summarized in Table 1. Patients with bi- and unilateral conductive hearing loss were divided into a group that purchased CC-HA and a group that did not, alongside their age, sex, and pure-tone audiometry results, which were compared. There was only one patient with bilateral conductive hearing loss in the non-purchase group, and the values for each of the cases are shown. There was no significant difference between the purchase and non-purchase groups in terms of age, sex, or pure-tone audiometry results for unilateral conductive hearing loss. Statistical analysis could not be performed for bilateral conductive hearing loss because there was only one patient in the non-purchase group.

**Figure 1:**
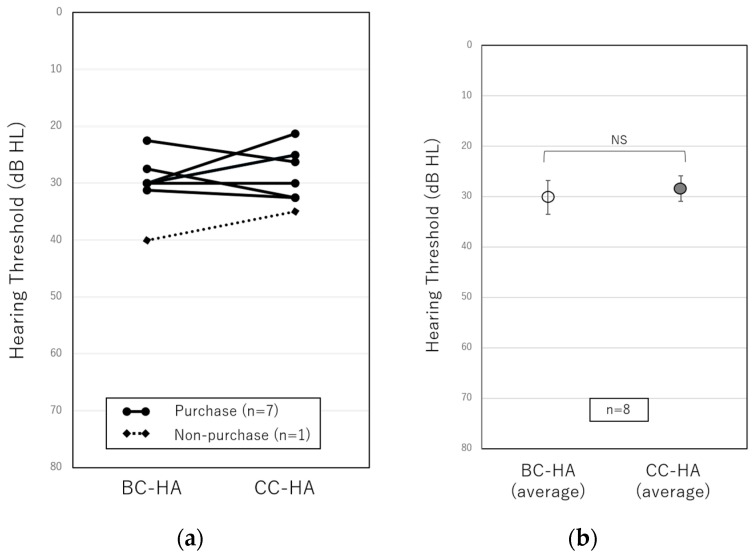
(**a**) Comparisons of individual hearing thresholds in sound field tests using the conventional BC-HA and the CC-HA. The solid line represents purchasers, and the dotted line represents non-purchasers. (**b**) Comparisons of the average hearing thresholds of eight patients using the BC-HA and the CC-HA. The error bars indicate standard errors. BC-HA: bone conduction hearing aid, CC-HA: cartilage conduction hearing aid, NS: not significant.

**Figure 2:**
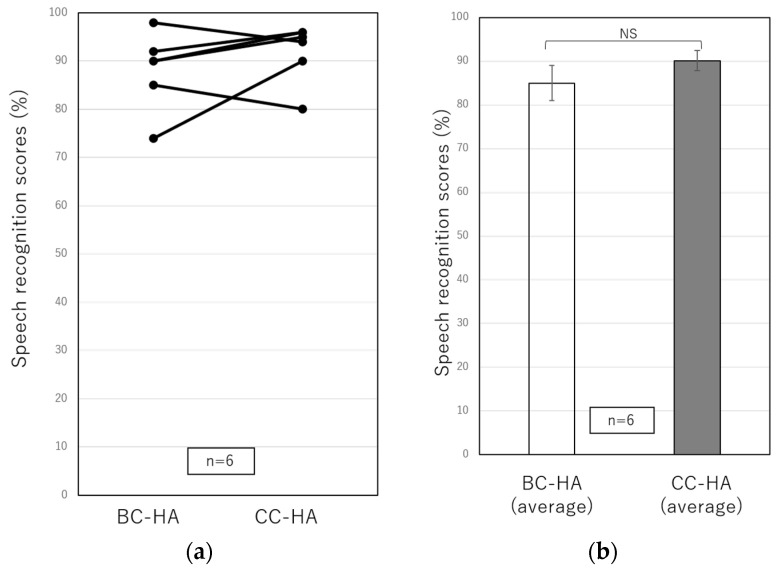
(**a**) Comparisons of individual speech recognition scores obtained from speech audiometry using the conventional BC-HA and the CC-HA. (**b**) Comparisons of the average SRSs of six patients using the BC-HA and the CC-HA. The error bars indicate standard errors. BC-HA: bone conduction hearing aid, CC-HA: cartilage conduction hearing aid, NS: not significant.

Figure 1 and Figure 2 show the results of the sound field test and speech audiometry for bilateral conductive hearing loss using conventional BC-HA and CC-HA. Those with circles at both ends and a solid line show the results for the purchase group, and those with squares at both ends and a dotted line show the results for the non-purchase group. In the sound field test in eight patients with bilateral conductive hearing loss (Figure 1), the results were 30.1 ± 1.7 dB for the BC-HA and 28.4 ± 1.6 dB for the CC-HA with no significant difference, indicating the non-inferiority using CC-HA. On the other hand, a comparison of the purchase and non-purchase groups using CC-HA could not be statistically analyzed because there was only one patient in the non-purchase group. In the speech audiometry in six patients with bilateral conductive hearing loss (Figure 2), the results were 88.1 ± 3.3% for the BC-HA and 91.8 ± 2.5% for the CC-HA, with no significant difference, indicating the non-inferiority of CC-HA in the speech audiometry as well as the sound field test.

**Figure 3:**
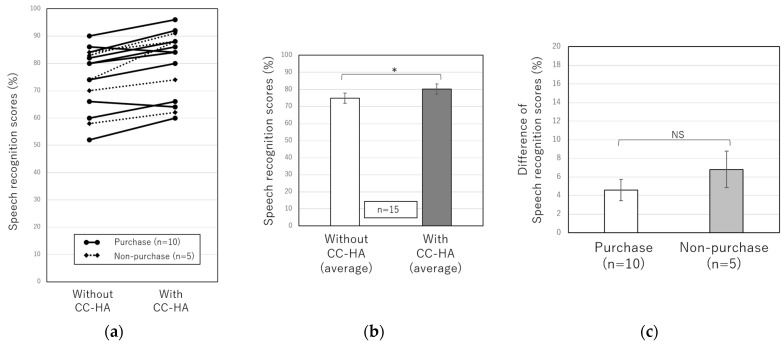
(**a**) Comparisons of individual speech recognition scores with and without the CC-HA at a sound pressure 10 dB lower than the sound pressure at which the highest SRSs were obtained without a hearing aid. The solid line represents purchasers, and the dotted line represents non-purchasers. (**b**) Comparisons of the average SRSs of 15 patients with and without the CC-HA. (**c**) Comparisons of SRSs between the purchasers and non-purchasers. The error bars indicate standard errors. NS: not significant, *: *p* < 0.05.

Figure 3 shows the speech audiometry results for 15 patients with unilateral conductive hearing loss who underwent the CC-HA hearing aid trial between January 2018 and October 2019. The average SRS in 15 patients with unilateral conductive hearing loss was 74.9 ± 3.0% without CC-HA, whereas it was 80.3 ± 3.1% while wearing CC-HA, showing a significant difference in speech clarity when using CC-HA (Figure 3b). However, the 15 patients were divided into the purchase and non-purchase groups, and the difference in the SRS with and without the CC-HA was compared between the two groups, but no significant difference was observed (Figure 3c).

**Figure 4:**
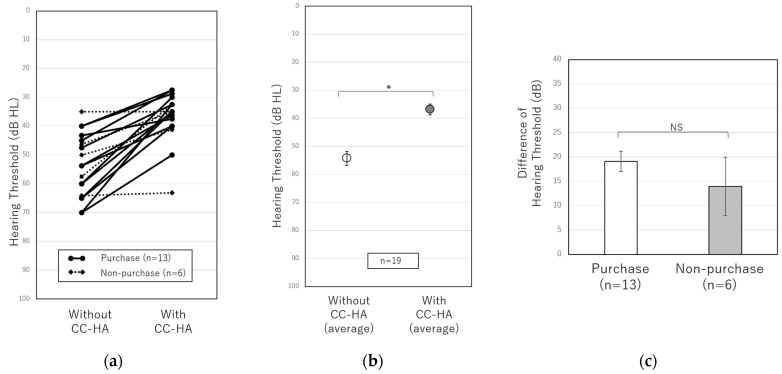
(**a**) Comparisons of individual hearing thresholds in sound field tests with and without CC-HA with 70 dB noise in the good ear. The solid line represents purchasers, and the dotted line represents non-purchasers. (**b**) Comparisons between the average hearing thresholds of 19 patients with and without the CC-HA. (**c**) Comparisons of hearing thresholds between the purchasers and non-purchasers. The error bars indicate standard errors. NS: not significant, *: *p* < 0.05.

**Figure 5:**
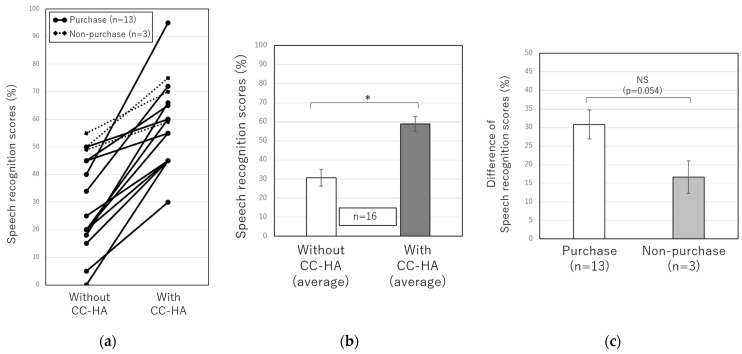
(**a**) Comparisons of individual speech recognition scores with and without the CC-HA with 70 dB noise in the good ear. The solid line represents purchasers, and the dotted line represents non-purchasers. (**b**) Comparisons of the average SRSs of 16 patients with and without CC-HA. (**c**) Comparisons of SRSs between the purchasers and non-purchasers. The error bars indicate standard errors. NS: not significant, *: *p* < 0.05.

Figure 4 and Figure 5 show the results of the sound field test and speech audiometry with and without CC-HA in 20 patients with unilateral conductive hearing loss who underwent the CC-HA hearing aid trial between October 2019 and December 2022. These tests were performed with 70 dB of noise in the good ear, as described above. The average of the sound field test in 19 patients with unilateral conductive hearing loss was 54.3 ± 2.5 dB without CC-HA and 36.8 ± 1.9 dB while wearing the CC-HA, showing a significant improvement while wearing CC-HA (Figure 4b). However, when the 19 patients were divided into the purchase and non-purchase groups, the difference in the sound field test results with and without CC-HA was compared between the two groups, and no significant difference was observed (Figure 4c). The average SRS in 16 patients with unilateral conductive hearing loss was 30.7 ± 4.4% without CC-HA and 58.9 ± 3.9% while wearing it, showing a significant improvement while using CC-HA (Figure 5b). When comparing the difference in SRS with and without CC-HA between the purchase and non-purchase groups, no significant difference was observed (*p* = 0.054); however, SRS tended to be better in the purchase group than in the non-purchase group when noise was produced in the good ear (Figure 5c).

## 4. Discussion

In this study, we first examined whether the test results differed between BC-HAs and CC-HAs in patients with bilateral conductive hearing loss. We did not observe a significant difference, and the average results of the sound field test and speech audiometry both improved slightly; therefore, CC-HA was shown to be non-inferior to BC-HA. Although not included in this study, there was one patient with bilateral conductive hearing loss who used an AC-HA as a conventional hearing aid, and CC-HA was as effective as the AC-HA in both sound field testing and speech audiometry. Although patients with bilateral conductive hearing loss were classified into purchase and non-purchase groups to compare the test results, there was only one non-purchaser; therefore, statistical analysis could not be performed. Further studies with more participants are needed to examine whether differences between CC-Has and BC-Has affect their willingness to use CC-HAs. Other differences between the BC-HA and the CC-HA may be perceived as comfort when wearing hearing aids. In fact, some patients who experienced wearing the BC-HA commented that after wearing the CC-HA for a while, skin pain and feelings of pressure were improved. Alternatively, some patients chose to wear the CC-HA with a simple transducer rather than the ear-chip-embedded transducer and felt that it was troublesome to stick them on. Furthermore, BC-HAs were often only worn in one ear for aesthetic reasons because if they were worn in both ears, the crimping feeling became stronger. In contrast, CC-HAs are often worn on both ears for comfort. Reeder et al. [17] reported that there was little difference in the hearing performance in silence when comparing unilateral hearing loss patients and those with normal hearing; however, in noisy conditions, it was significantly reduced in patients with unilateral hearing loss. Bagatto et al. [18] reported the benefits of binaural hearing. In fact, among the participants of this study, six out of seven patients with bilateral conductive hearing loss who purchased CC-HAs wore them in both ears. Based on this, those who choose CC-HAs tended to choose to wear them in both ears, and the fact that they could be worn in both ears may also lead to a willingness to wear CC-HAs.

Next, we investigated whether the binaural hearing effect of wearing CC-HA improved speech recognition in patients with unilateral conductive hearing loss. First, we compared SRS with and without CC-HA at a sound pressure 10 dB lower than the sound pressure at which the highest SRSs were obtained without a hearing aid. As a result, an improvement in the SRS was observed when wearing CC-HA. This result was similar to the result of the study reported by Akasaka et al. [16], who examined the binaural hearing effect of CC-HA in patients with unilateral aural atresia. However, in our study, the average difference with and without the CC-HA was small (approximately 5%), and when the patients with unilateral conductive hearing loss were divided into the purchase and non-purchase groups, there was no significant difference in the improvement of SRS between the two groups. Therefore, we modified the testing method and performed a sound field test and speech audiometry with 70 dB of noise in the good ear. In this method, the noise masked the good ear, thus confirming the effectiveness of wearing CC-HA on the affected side. The results of the sound field test and speech audiometry significantly improved with the use of CC-HA, and some patients commented on the effect of wearing CC-HA. On the other hand, when we compared the patients in the purchase and non-purchase groups, no significant differences were found in the results of sound field and speech audiometry under noise. However, there was a tendency for the purchase group to perform better than the non-purchase group on speech audiometry with noise in the good ear. Since the number of cases was small, further investigation is necessary, but it is possible that feeling the effect of wearing the CC-HA on speech recognition may affect patients’ willingness to wear it. In addition, regarding the sound field test under noise, the noise was set at 70 dB to avoid excessive discomfort to the good-hearing ear. Therefore, it was possible that some patients with a higher threshold on the affected side did not correctly obtain the sound field hearing on the affected side before wearing CC-HA, resulting in no difference between the purchase and non-purchase groups, and further study design was considered necessary.

In addition, the economic situation may also influence the willingness to wear CC-HAs because of their high cost. When introducing CC-HAs to patients, it is important to provide appropriate information not only about their effect on hearing but also about the comfort and aesthetics of CC-HAs, as well as the cost of purchasing CC-HAs and the available subsidies.

The limitations of this study included the low number of non-purchasers, which resulted in an imbalance between purchasers and non-purchasers. In particular, there was only one non-purchaser among the patients with bilateral conductive hearing loss, which was considerably low. It is not possible to perform a statistical analysis separating the CC-HA purchasers and non-purchasers. The acquisition of additional participants was, therefore, necessary. Additionally, because of the low number of non-purchasers, it was thought that patients who were not proactive in purchasing were not very cooperative in examinations, and there were cases where data could not be obtained, suggesting the presence of selection bias. Furthermore, there were many young patients with microtia at our hospital. As a result, confounding factors such as the presence or absence of subsidies for purchases may have been at play.

## 5. Conclusions

This study examined the effectiveness of CC-HA in patients with bi- and unilateral conductive hearing loss and investigated the predictive factors that lead to their willingness to wear CC-HA. The results indicate that CC-HA was non-inferior to BC-HA in patients with bilateral conductive hearing loss, and its usefulness was demonstrated in patients with unilateral conductive hearing loss. In patients with unilateral conductive hearing loss, good results on speech audiometry while wearing CC-HA with noise in the good ear may influence their willingness to wear CC-HA.

## Figures and Tables

**Table 1:** Characteristics of patients.

Bilateral Conductive Hearing Loss					
	N	Age (Year, Mean ± SD)	Female/Male	PTA in Poorer Hearing Ear (dB HL, Mean ± SD)	PTA in Better Hearing Ear (dB HL, Mean ± SD)

Purchase	7	15.5 ± 2.0	5/2	72.7 ± 8.0	66.4 ± 16.1
Non-purchase	1	6	1/0	78.8	76.3
**Unilateral Conductive Hearing Loss**					
	**N**	**Age** **(Year, Mean ± SD)**	**Female/Male**	**PTA in Poorer Hearing Ear** **(dB HL, Mean ± SD)**	**PTA in Better Hearing Ear** **(dB HL, Mean ± SD)**

Purchase	24	16.3 ± 13.2	9/15	78.9 ± 10.4	10.2 ± 6.0
Non-purchase	11	13.3 ± 6.6	6/5	72.7 ± 12.7	11.6 ± 3.6

PTA: pure-tone average of thresholds at 500, 1000, and 2000 Hz. Purchase: a group that purchased CC-HA. Non-purchase: a group that did not purchased CC-HA.

## Data Availability

Not applicable.
